# Supplementary material for: The impact of epidemic infectious diseases on the relationship between subjective well-being and social class identity in older adults: The mediating role of Self-rated health
Source: PLoS One. 2024 Mar 26;19(3):e0301289. doi: 10.1371/journal.pone.0301289 (PMC10965052; doi:10.1371/journal.pone.0301289)
Supplement: S1 File — (DOCX) [file pone.0301289.s001.docx]

| Variable | 2018 | | | | 2021 | | | |
| --- | --- | --- | --- | --- | --- | --- | --- | --- |
|  | Mean | Std. dev. | Min | Max | Mean | Std. dev. | Min | Max |
| subjective well-being | 3.961 | 0.828 | 1 | 5 | 4.059 | 0.850 | 1 | 5 |
| Social Class Identity | 4.159 | 1.721 | 1 | 10 | 4.291 | 2.010 | 1 | 10 |
| health | 3.118 | 1.052 | 1 | 5 | 3.110 | 1.112 | 1 | 5 |
| gender | 1.521 | 0.500 | 1 | 2 | 1.499 | 0.500 | 1 | 2 |
| census | 1.495 | 0.500 | 1 | 2 | 1.426 | 0.495 | 1 | 2 |
| education | 2.498 | 1.283 | 1 | 7 | 2.530 | 1.175 | 1 | 7 |
| ln-income | 9.456 | 1.452 | 4.605 | 15.42 | 10.14 | 2.357 | 3.912 | 16.12 |
| trust | 3.702 | 0.955 | 1 | 5 | 3.843 | 0.977 | 1 | 5 |
| fair | 3.326 | 1.017 | 1 | 5 | 3.612 | 0.999 | 1 | 5 |

|  | (1) | (2) | (3) |
| --- | --- | --- | --- |
|  | happiness | health | happiness |
| main |  |  |  |
| stratum | 0.172*** | 0.090*** | 0.159*** |
|  | (0.012) | (0.011) | (0.012) |
|  |  |  |  |
| gender | 0.101** | -0.104** | 0.121** |
|  | (0.038) | (0.036) | (0.039) |
|  |  |  |  |
| census | 0.027 | -0.054 | 0.037 |
|  | (0.056) | (0.052) | (0.056) |
|  |  |  |  |
| education | 0.012 | 0.065*** | -0.000 |
|  | (0.018) | (0.017) | (0.018) |
|  |  |  |  |
| logincome | 0.090*** | 0.100*** | 0.072*** |
|  | (0.020) | (0.018) | (0.020) |
|  |  |  |  |
| trust | 0.149*** | 0.025 | 0.147*** |
|  | (0.020) | (0.019) | (0.020) |
|  |  |  |  |
| fair | 0.329*** | 0.056** | 0.325*** |
|  | (0.020) | (0.018) | (0.020) |
|  |  |  |  |
| health |  |  | 0.193*** |
|  |  |  | (0.019) |
| / |  |  |  |
| cut1 | 0.758*** | -0.159 | 1.079*** |
|  | (0.184) | (0.165) | (0.188) |
|  |  |  |  |
| cut2 | 1.704*** | 0.957*** | 2.048*** |
|  | (0.179) | (0.165) | (0.183) |
|  |  |  |  |
| cut3 | 2.374*** | 1.754*** | 2.735*** |
|  | (0.180) | (0.166) | (0.184) |
|  |  |  |  |
| cut4 | 4.267*** | 2.990*** | 4.660*** |
|  | (0.187) | (0.169) | (0.192) |
| N | 3717 | 3717 | 3717 |
| R2 | 0.1007 | 0.0251 | 0.1136 |
|  | (1) | (2) | (3) |
|  | happiness | health | happiness |
| main |  |  |  |
| stratum | 0.172*** | 0.090*** | 0.159*** |
|  | (0.012) | (0.011) | (0.012) |
|  |  |  |  |
| gender | 0.101** | -0.104** | 0.121** |
|  | (0.038) | (0.036) | (0.039) |
|  |  |  |  |

|  | (1) | (2) | (3) |
| --- | --- | --- | --- |
|  | happiness | health | happiness |
| main |  |  |  |
| stratum | 0.123^***^ | 0.096^***^ | 0.110^***^ |
|  | (0.015) | (0.014) | (0.016) |
|  |  |  |  |
| gender | -0.071 | -0.132^*^ | -0.052 |
|  | (0.060) | (0.055) | (0.060) |
|  |  |  |  |
| census | 0.175^*^ | 0.103 | 0.159^*^ |
|  | (0.068) | (0.063) | (0.068) |
|  |  |  |  |
| education | 0.010 | 0.104^***^ | -0.005 |
|  | (0.029) | (0.026) | (0.029) |
|  |  |  |  |
| lnincome | 0.006 | 0.018 | 0.003 |
|  | (0.013) | (0.012) | (0.013) |
|  |  |  |  |
| trust | 0.191^***^ | -0.015 | 0.195^***^ |
|  | (0.031) | (0.029) | (0.031) |
|  |  |  |  |
| fair | 0.327^***^ | 0.086^**^ | 0.318^***^ |
|  | (0.031) | (0.029) | (0.031) |
|  |  |  |  |
| health |  |  | 0.149^***^ |
|  |  |  | (0.028) |
| / |  |  |  |
| cut1 | 0.161 | -0.396 | 0.455 |
|  | (0.235) | (0.209) | (0.242) |
|  |  |  |  |
| cut2 | 0.885^***^ | 0.484^*^ | 1.190^***^ |
|  | (0.225) | (0.209) | (0.233) |
|  |  |  |  |
| cut3 | 1.523^***^ | 1.360^***^ | 1.837^***^ |
|  | (0.226) | (0.211) | (0.234) |
|  |  |  |  |
| cut4 | 3.276^***^ | 2.380^***^ | 3.615^***^ |
|  | (0.235) | (0.214) | (0.245) |
| *N* | 1544 | 1544 | 1544 |
| *R*^2^ | 0.0938 | 0.0276 | 0.1024 |

Bootstrap results Number of obs = 3,717

Replications = 1,000

Command: sgmediation SWB, mv( health ) iv( SocialClassIdentity ) cv( gender education lnincome

trust fair census )

_bs_1: r(ind_eff)

_bs_2: r(dir_eff)

_bs_3: r(tot_eff)

------------------------------------------------------------------------------

| Observed Bootstrap Normal-based

| coefficient std. err. z P>|z| [95% conf. interval]

-------------+----------------------------------------------------------------

_bs_1 | .0104127 .0017987 5.79 0.000 .0068874 .013938

_bs_2 | .0991854 .0082149 12.07 0.000 .0830846 .1152862

_bs_3 | .1095981 .0084627 12.95 0.000 .0930115 .1261847

------------------------------------------------------------------------------

Bootstrap results Number of obs = 1,544

Replications = 1,000

Command: sgmediation SWB, mv( health ) iv( SocialClassIdentity ) cv( gender education lnincome

trust fair census )

_bs_1: r(ind_eff)

_bs_2: r(dir_eff)

_bs_3: r(tot_eff)

------------------------------------------------------------------------------

| Observed Bootstrap Normal-based

| coefficient std. err. z P>|z| [95% conf. interval]

-------------+----------------------------------------------------------------

_bs_1 | .0097274 .002441 3.99 0.000 .0049432 .0145116

_bs_2 | .0682463 .0112458 6.07 0.000 .046205 .0902876

_bs_3 | .0779737 .0112626 6.92 0.000 .0558994 .100048

------------------------------------------------------------------------------
