# Supplementary material for: The impact of epidemic infectious diseases on the relationship between subjective well-being and social class identity in older adults: The mediating role of Self-rated health
Source: PLoS One. 2024 Mar 26;19(3):e0301289. doi: 10.1371/journal.pone.0301289 (PMC10965052; doi:10.1371/journal.pone.0301289)
Supplement: S2 File — (ZIP) [file pone.0301289.s002.zip › Supporting Information/Introduction.docx]

Introduction

Please open the dataset with Stata software.
