# Supplementary material for: The impact of epidemic infectious diseases on the relationship between subjective well-being and social class identity in older adults: The mediating role of Self-rated health
Source: PLoS One. 2024 Mar 26;19(3):e0301289. doi: 10.1371/journal.pone.0301289 (PMC10965052; doi:10.1371/journal.pone.0301289)
Supplement: Attachment — Submitted filename: Response to Reciewers.docx [file pone.0301289.s004.docx]

Dear Editor,

Thank you for giving us an opportunity to revise our manuscript, we appreciate editor and reviewers very much for their positive and constructive comments and suggestions on our manuscript entitled “The impact of epidemic infectious diseases on the relationship between subjective well-being and social class identity in older adults: the mediating role of Self-rated health”(PONE-D-23-34398). We have studied the comments carefully and have made revision in this paper. The responses to the comments from three reviewers are offered separately, and the corresponding modification position page in the revised manuscript can be found in the rightmost column of the response table blow. Moreover, the revised paragraphs (sentences) are labeled with revision mode.

The paragraph or sentence revised according to the REVIEWER is represented by a revision pattern.

**Respond to Reviewer**

**Introduction**

**•	Elaborate more on the broader significance and implications of the study in the context of healthy aging. Highlight the potential contributions of the research to the existing literature and how it addresses gaps in understanding the relationship between SWB, social class identity, and Self-rated health in older adults.**

**•	To enhance the quality of your introduction, consider incorporating the following references**

Thanks to the advice of the expert teachers, I have enriched the citation of this paper according to your comments and have cited several references that you have recommended as highly relevant according to the content and purpose of the study.

**Method:**

**•	Consider including the reliability or validity measures of the chosen items or questions used to measure subjective well-being, social class identity, and Self-rated health. This adds credibility to the chosen instruments.**

Thank you for the expert teacher's opinion. CGSS is a large-scale nationwide social sample survey jointly initiated by Renmin University of China and Hong Kong University of Science and Technology, with the main purpose of understanding the living and employment conditions of urban and rural residents in China as well as their attitudes towards hot issues in the society. The survey aims to explore the living conditions, employment situation and attitudes towards hot issues in society of urban and rural residents. The survey covers 30 provincial-level administrative regions in China, which reflects the real situation of China's society in a more comprehensive and integrated way, and has a certain degree of scientificity and representativeness. Moreover, the CGSS questionnaire has been carefully designed and strictly controlled, and has passed the reliability and validity test, so the questionnaire is scientific and authoritative.

**•	Clarify how missing data were handled, particularly in the exclusion criteria. Specify if imputation techniques were used or if cases with missing values were excluded from the analysis**

Thank you for your comments, I have added and modified them as you requested. Thank you for your comments, I have added and modified them as you requested, see line 176.

**Discussion**

**1.	When referring to theoretical frameworks such as Maslow's Hierarchy of Needs theory or social-emotional choice theory, provide a brief explanation of these theories for readers who may not be familiar with them**

Thank you for your valuable comments, and I have added a brief description of the relevant theories, as shown in lines 305 and 339.

**2.	When discussing the temporal comparisons between 2018 and 2021, elaborate on the reasons behind the changes observed. Discuss potential societal or economic factors that may have influenced the increase in economic income, SWB, and social class identity.**

Thank you for your suggestion, I had followed your suggestion to analyze the reason behind the change, see line 371.
